# “Discordant Airflow Obstruction: GOLD versus LLN”

**DOI:** 10.1155/2012/751692

**Published:** 2012-08-07

**Authors:** Martin R. Miller

**Affiliations:** Institute of Occupational and Environmental Medicine, University of Birmingham, Birmingham B15 2TT, UK

I recently came across the paper of Lamprecht et al. “*Subjects with discordant *airways obstruction: lost between spirometric definitions of COPD” which deals with people who are defined as having COPD by the GOLD criteria [1] but not by using a lower limit of normal (LLN) method. The authors found this discordant group had fewer symptoms and less severe FEV_1_ impairment than those diagnosed with obstruction by both methods, and so the discordant group did not have clinically significant airway obstruction. However, they conclude that this discordant group had a clinical profile characterised by relevant comorbid disease, namely, more heart disease. The analysis they present does not allow for their conclusion that the GOLD criteria on lung function in some way specially identifies this risk, because the authors failed to account for other relevant covariates in their analysis. The discordant group is known to be biased towards men and older subjects by the way the fixed ratio of FEV_1_/FVC < 0.7 discriminates against them versus the LLN [2]. In their analysis the authors have accounted for the age differences between the groups but not for the observed sex differences; namely, men accounted for 81.5% of the discordant group but were only 48.7% of the concordant group. Since heart disease is more prevalent in men, the authors' results cannot be used to suggest that identifying their discordant group from lung function testing in some way specially identifies those at risk of heart disease. It just introduces a male bias.
